# Contralateral migration of oculomotor neurons is regulated by Slit/Robo signaling

**DOI:** 10.1186/s13064-016-0073-y

**Published:** 2016-10-22

**Authors:** Brielle Bjorke, Farnaz Shoja-Taheri, Minkyung Kim, G. Eric Robinson, Tatiana Fontelonga, Kyung-Tai Kim, Mi-Ryoung Song, Grant S. Mastick

**Affiliations:** 1 Department of Biology, University of Nevada, Reno, NV 89557 USA; 2 School of Life Sciences, Gwangju Institute of Science and Technology, Oryong-dong, Buk-gu, Gwangju 500-712 Republic of Korea

**Keywords:** Oculomotor, Motor neuron, Migration, Floor plate, Slit/Robo

## Abstract

**Background:**

Oculomotor neurons develop initially like typical motor neurons, projecting axons out of the ventral midbrain to their ipsilateral targets, the extraocular muscles. However, in all vertebrates, after the oculomotor nerve (nIII) has reached the extraocular muscle primordia, the cell bodies that innervate the superior rectus migrate to join the contralateral nucleus. This motor neuron migration represents a unique strategy to form a contralateral motor projection. Whether migration is guided by diffusible cues remains unknown.

**Methods:**

We examined the role of Slit chemorepellent signals in contralateral oculomotor migration by analyzing mutant mouse embryos.

**Results:**

We found that the ventral midbrain expresses high levels of both Slit1 and 2, and that oculomotor neurons express the repellent Slit receptors Robo1 and Robo2. Therefore, Slit signals are in a position to influence the migration of oculomotor neurons. In Slit 1/2 or Robo1/2 double mutant embryos, motor neuron cell bodies migrated into the ventral midbrain on E10.5, three days prior to normal migration. These early migrating neurons had leading projections into and across the floor plate. In contrast to the double mutants, embryos which were mutant for single *Slit* or *Robo* genes did not have premature migration or outgrowth on E10.5, demonstrating a cooperative requirement of Slit1 and 2, as well as Robo1 and 2. To test how Slit/Robo midline repulsion is modulated, we found that the normal migration did not require the receptors Robo3 and CXCR4, or the chemoattractant, Netrin 1. The signal to initiate contralateral migration is likely autonomous to the midbrain because oculomotor neurons migrate in embryos that lack either nerve outgrowth or extraocular muscles, or in cultured midbrains that lacked peripheral tissue.

**Conclusion:**

Overall, our results demonstrate that a migratory subset of motor neurons respond to floor plate-derived Slit repulsion to properly control the timing of contralateral migration.

## Background

The oculomotor neurons are the most anterior motor neurons in the CNS, forming the oculomotor nerve (nIII). Their axons emerge from the ventral midbrain and innervate four of the six extraocular muscles. Development of the oculomotor system must occur with spatial and temporal accuracy to properly position the motor neuron cell bodies and to guide their axons to corresponding extraocular muscles. Errors in development can lead to abnormal eye movements or alignment, termed strabismus, and may result in partial blindness reviewed in [1]. The mechanisms that guide the development of the oculomotor system remain poorly understood.

Early in embryonic development, clusters of oculomotor neurons project axons ipsilaterally toward muscle targets, similar to most motor neurons. However, during extraocular muscle innervation, a subset of neurons in the oculomotor nucleus repolarize to send a second process into and across the midline. This subset of motor neuron cell bodies then migrate across the ventral midbrain with axons trailing to join the contralateral oculomotor nucleus [2–7]. This process generates the oculomotor commissure and connects motor neurons located in the caudal half of the oculomotor nucleus to the contralateral superior (dorsal) rectus muscle. Contralateral innervation of the superior rectus muscle is highly conserved among vertebrates [8, and references within].

Oculomotor neurons navigate across the embryonic midline independent of an identifiable glial scaffold, and it was suggested that a “diffusible substance” guides the migrating neurons across the midline [3]. The embryonic ventral midline, consisting of specialized floor plate tissue, is a source of diffusible guidance factors [9]. However, it is unknown whether floor plate guidance cues guide oculomotor neurons. We have focused on two diffusible guidance factors that regulate midline crossing of commissural axons, the Slit proteins and Netrin1. In the developing spinal cord and hindbrain, Slits and Netrin1 mediate migration across the floor plate by their opposing chemotactic actions. The Slit proteins repel both navigating axons and migrating neuron cell bodies reviewed in [10, 11]. In vertebrates, there are three Slit proteins, of which Slit1 and 2 act at the midline to repel axons that express the Slit receptor Robo1 or 2 [12–14]. The third Slit receptor, Robo3 may either counteract the repellent activity of Robo1 and 2 [15] or facilitate Netrin1 attraction [16]. In contrast with Slit signaling, Netrin1 attracts both axons and neurons toward the midline thorough the receptor Deleted in Colorectal Cancer (DCC) [17–21]. Importantly, prior studies in hindbrain showed that these midline guidance signals are important for positioning other cranial neurons, including cranial axon repulsion by Netrin1 [22], and facial branchiomotor migration defects in Slit and Robo mutants and Netrin mutants [23, 24]. Both Slits and Netrin1 are expressed at the ventral midline in the midbrain during early developmental stages [25, 26]. The expression of both Slits and Netrin1 in the developing midbrain, coupled with their role in guiding midline crossing of axons, suggests a role for these cues in guiding the midline migration of oculomotor neurons.

Here we describe how Slit, but not Netrin, acts to gate migration of oculomotor cell bodies into the floor plate. We show that the initial clusters of motor neurons are in fact not static, but have considerable migratory potential, which is demonstrated by abnormal early migration across the floor plate when Slit/Robo signaling is disrupted.

## Methods

### Mouse embryos

#### Ethics approval

Animal experiments were approved by the UNR IACUC, following NIH guidelines, with the approved protocol #2015-00435. DCC embryos are previously described [27]. Robo, Slit and Netrin1 mutant mice were a kind gift from Marc Tessier-Lavigne (Rockefeller), and Frederic Charron (ICMR, Montreal CA). Mating to obtain various Slit mutant combinations was previously described [13]). Robo, Slit and Netrin1 PCR genotyping were performed as previously described [12, 27–29]. CXCR4 mutant embryos were a gift from John Rubenstein (UCSF). Pitx2 mutant embryos were a kind gift from Philip J Gage (University of Michigan, Ann Arbor). Images of Robo3 mutant embryos were provided by Alain Chedotal (INSERM, Paris). Wild type CD-1 mice were purchased from Charles River Laboratories (Wilmington, MA USA). Embryos were collected in the afternoon of day 10.5, 13.5, or 16.5 with embryonic day 0.5 designated as the day of the vaginal plug. Embryos were fixed with 4 % paraformaldehyde (PFA) overnight or for several days. E16.5 embryos were fixed via cardiac perfusion, and fixed overnight in 4 % PFA.

### In situ hybridization

Whole mount in situ hybridization was previously described [30]. Probes for *Slit1,Slit2, Slit3, Robo1, Robo2, Robo3* were a kind gift from Marc Tessier-Lavigne, (Rockefeller).

### Immunohistochemistry

CD1 E10.5 and E13.5 embryos were dissected in cold 0.4 M phosphate buffer, and fixed with 0.4 % PFA for 1 day. Embryos were then embedded for cryostat sectioning as described [13]. 20 um cryostat sections were rinsed in warm 0.4 % phosphate buffer, and washed with PBS with 0.1 % TritonX-100, and 10 % normal goat serum (PBST). Primary antibodies were diluted in PBST and were applied overnight at room temperature. Primary antibodies included Robo3 (anti-rabbit, Abcam) 1:200, β-galactosidase (Jackson) 1:10,000, Islet1/2 ( DHSB, 39.4D5) 1:200, Robo1 and Robo2 antibodies (kind gift from Elke Stein, Yale; validated in [31]) 1:10,000. Sections were washed several times in PBST and Secondary antibody was applied. Secondary antibodies (Alexa 488, Alexa 555) were diluted in PBST and used at 1:200 for one hour at room temperature. For whole mount labeling of Islet1/2, tissue was placed in primary antibody for 4 days diluted in PBST. The biotin-avidin system (Invitrogen) was used for Islet1/2 amplification, biotin (donkey anti-mouse, 1:100 in PBST) was applied overnight at 4°, washed overnight in PBST, and followed by avidin555 or 488 (1:200in PBST) overnight at 4°.

### Axon tracing

To back-label the oculomotor nucleus and midline crossing fibers, the lipophilic dye, DiI, red, or DiO, green, was crushed onto the oculomotor nerve. First, the skin and mesenchymal tissue was carefully removed from the cephalic flexure ventral to the midbrain to reveal the nIII nerve, then forceps were used to pinch a small crystal of dye onto the nerve. Embryos were placed in 4 % PFA with .1 % EDTA at 37 °C overnight (E10.5) or up to 3 days (E13.5, E16.5) to allow dye to travel. To visualize the labeled oculomotor nucleus, a 200 um coronal section was cut with a vibratome, and imaged with a confocal microscopy. To visualize the anterior-posterior axis of the OM nucleus, the embryo was cut along the dorsal edge to reveal the midline (open book preparation).

### Quantification of motor neurons in the floorplate

E10.5 midbrains were dissected from various combinations of Slit and Robo mutant mice and antibody labeled for Islet1/2 in open book or sectioned preparations. Islet-positive cells that were located in the space between the two defined oculomotor nuclei were quantified. The average number of cells was graphed with standard deviation indicated by error bars. Significance was determined by Tukey HSD one-way-ANOVA.

### Explant cultures of isolated midbrain tissue

E11.5 mouse midbrain tissues were dissected away from peripheral tissues as an open book preparation, and were cultured in a three-dimensional collagen gel matrix. The cultured tissues were fixed in 4 % PFA after 0, 24, 48, and 72 h of incubation. To label the migrated neurons, explant tissues were washed in PBS containing 10 % FBS and 1 % Triton for several hours (PBST). Primary antibody (1:200 mouse anti-Islet1/2, DSHB) in PBST was applied for 3-4 days. After washing the tissues for several hours, secondary antibody (1:200 Cy3 anti-rabbit (Invitrogen)) in PBST was applied for 2-3 days. The tissues were washed again and mounted on the slides for image acquisition under the confocal microscope (Olympus FV10-ASW).

## Results

### A subset of oculomotor neurons migrate across the midline of the midbrain

To investigate contralateral migration of oculomotor neurons in wild type mice, we first determined the time course of normal migration. To distinguish cell bodies and projections that originate from the left or right oculomotor nuclei, we back-labeled the right and left nucleus with DiI and DiO crushed onto the right and left nIII, respectively. We refer to cells and axons labeled through nIII in this manner as oculomotor, although we note that the embryonic nIII also includes visceral motor fibers from the closely-associated Edinger-Westphal nucleus. To determine the location of oculomotor neuron cell bodies, we labeled the ventral midbrain with Islet1/2 antibody, a general motor neuron marker [32].

**Fig. 1:**
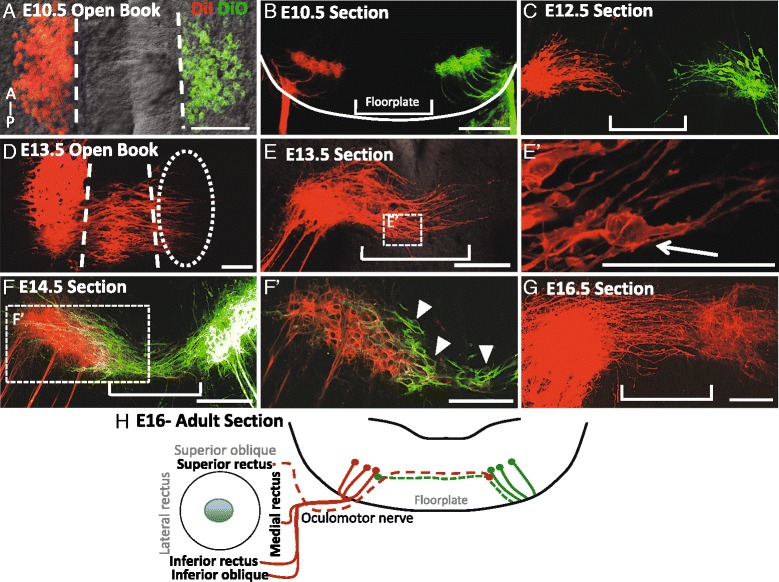
The oculomotor commissure is generated from E12.5 to E16.5 in the ventral midbrain. Oculomotor nuclei were back-labeled with peripheral application of DiI to the right oculomotor nerve (*red*) and DiO to the left oculomotor nerve (*green*) in mouse embryos on E10.5-16.5. The labeling is shown as either open book preparations revealing the anterior–posterior length of the oculomotor nucleus (**A**, **D**) or transverse sections of the midbrain (**B**, **C**, **E**, **F**, **G**), **A**, **B**. On E10.5, all oculomotor cell bodies were located on either side of the floor plate (**A**), ipsilateral to their nerve (**B**). **C** On E12.5, leading processes projected into the floor plate. **D** On E13.5, leading processes projected from the posterior half of the oculomotor nuclei across the midline toward the contralateral oculomotor nucleus. **E** Apparent cell bodies were located within the numerous leading processes within the floor plate (E’). **F** On E14.5, leading processes have crossed the floor plate to contact the contralateral nucleus. F’. In single focal planes by microscopy, contralateral cell bodies were located on the ventromedial aspect of the opposing nucleus as well as in the floor plate (*arrow heads* point to cell bodies outlined in *green*). **G** On E16.5 no cell bodies were located in the floor plate, and leading processes spanned the contralateral nucleus. **H** Schematic showing that the superior rectus extraocular muscle is innervated by contralateral oculomotor neurons and their midline axon fibers (*dashed lines*). Scale bars, 100 μm

On E10.5, oculomotor neurons reside ipsilateral to their respective peripheral nerves (Fig. 1A, B), clustered at the edge of the floor plate (Fig. 1B). Later in development, on E12.5 and 13.5, cell bodies located within the caudal half of the oculomotor nucleus extended labeled processes toward the midline (Fig. 1C, D). In agreement with previous findings in chick and rat, the leading tips of these processes were generally compact with one leading tip [2, 6]. These processes initially projected toward the ventricular surface of the neural tube, then curved slightly down toward the ventral midline and crossed the floor plate to the contralateral oculomotor nucleus (Fig. 1C). By E13.5, leading processes reached the ventrolateral region of the contralateral nucleus and numerous cell bodies were outlined by the lipophilic dye in the floorplate, with a concentration in the midline. By E14.5 leading fibers intercalated into the contralateral nucleus with cell bodies reaching the ventromedial aspect of the nucleus (arrow heads in Fig. 1F'). While the cell bodies accumulated at this ventromedial position, surprisingly many leading processes extended through and past the contralateral nucleus, suggesting that the trailing cell bodies encounter stop cues distinct from the leading processes. The commissure linking the bilateral oculomotor nuclei appeared complete by E16.5, with crossing axons forming the commissure but no cell bodies visible within it (Fig. 1G and schematic Fig. 1H).

**Fig. 2:**
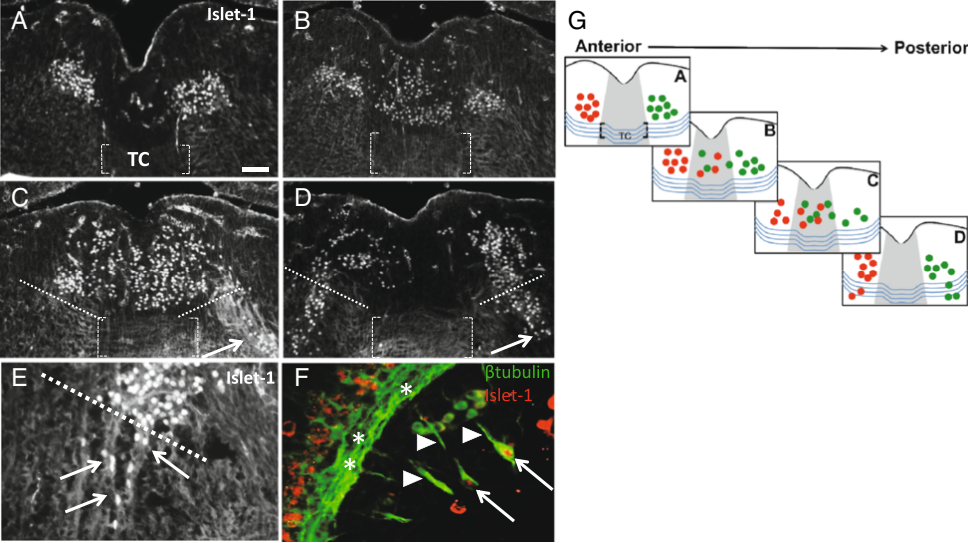
On E13.5, Islet 1/2 positive neurons migrate across the midline independent of the existing commissure, with a small number of Islet positive neurons found in fibers projecting away from the nucleus. **A**-**D**. Transverse sections through the oculomotor nucleus on E13.5, shown anterior (**A**) to posterior (**D**), were antibody labeled for the motor neuron-specific transcription factor Islet1/2. The ventral tegmental commissure traveling through the floor plate is indicated (*brackets* in **A**-**D**). **A** In anterior sections, Islet1/2+ oculomotor neurons were located in distinct nuclei on either side of the floor plate, with no midline cell bodies visible (only non-specific blood vessel labeling is seen in the midline). **B**-**D** Large numbers of oculomotor neurons were located in the floor plate in a distinct stream above the commissure in intermediate (**B**) through posterior sections (**C**, **D**). In posterior sections, a subset of Islet1/2+ cells formed a stream toward the pial surface, moving outside of the bounds of the nucleus (marked with *dashed white lines*), apparently along the nerve fibers projecting to the ventral exit point (*arrows* in **C**, **D**). **E** Islet 1/2+ neurons were located in fibers projecting from the oculomotor nucleus toward the pial surface of the neural tube (*arrows* point to fibers projecting away from the oculomotor nucleus). **F** Sagittal section through the oculomotor nerve, projecting from ventral exit points (*asterisks*) toward the eye (*arrow head* points to peripheral oculomotor nerve fibers), shows Islet 1/2 positive cells located within the peripheral oculomotor nerve (*arrows* in **F**). **G** Schematic indicating the location of Islet1/2+ cell bodies in anterior to posterior sections. Right and left oculomotor nuclei are indicated by *green* and *red* colors respectively. The tegmental commissure is shown as *blue curved lines* traveling through the floor plate (*gray color*). Islet positive cells are located above the commissure. Abbrev: Tegmental commissure (TC). Scale bar100 μm

To define the anterior-posterior organization of midline crossing on E13.5, transverse sections through the oculomotor nucleus were labeled with the motor-neuron specific marker Islet1/2. In sections taken through the anterior portion of the oculomotor nucleus, motor neurons bundled in discrete nuclei adjacent to the floor plate (Fig. 2A). However, in sections ranging from the middle to caudal oculomotor nucleus, several motor neuron cell bodies were separated from the nuclei, and found within the floor plate (Fig. 2B, C). Cell bodies in the floor plate did not contact the existing ventral tecto-tegmental commissure at the pial surface (brackets in Fig. 2A-D). Instead, the neurons migrated in the ventricular half of the tissue to pioneer a distinct commissure. Interestingly, cell bodies were also observed within fibers that project from the oculomotor nucleus toward the nerve exit points (arrow in Fig. 2C, D, E). Previous research noted cell bodies located within the peripheral oculomotor nerve [33]. We found that cells located within the peripheral oculomotor nerve expressed Islet1/2 (arrow heads point to nIII fibers, arrows point to Islet1/2 + cells in Fig. 2F). These images suggest that Islet1/2 positive cells migrating away from the oculomotor nucleus make their way into the peripheral nerve. Carpenter (1906) hypothesized that cells located in the peripheral nerve will migrate to join the ciliary ganglion [33].

### The Slit family of guidance cues and their receptors are in position to inhibit migration into the floor plate on E10.5

**Fig. 3:**
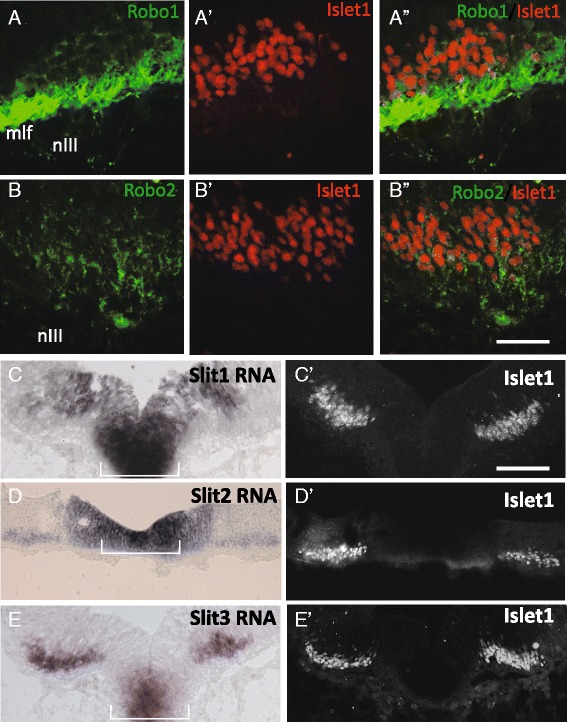
The guidance cues Slit1 and 2, and receptors Robo1 and 2, are in position to prevent oculomotor migration across the floor plate on E10.5. Coronal sections were taken through the posterior midbrain on E10.5 to determine Slit and Robo expression. Following protein (Robos) or mRNA (Slits) labeling, the same or adjacent section was labeled for Islet1/2 to co-localize expression to oculomotor neurons (A’-B’, A”, B”). **A**, **B** Robo1 and 2 protein was found in the ventral midbrain and in the oculomotor nerve fibers (nIII). Expression was co-localized with Islet1/2 indicating Robo1 and Robo2 expression in oculomotor neurons (A”,B”). Robo1 antibody also strongly labels the adjacent medial longitudinal fasciculus (mlf). **C**-**E** In situ hybridization for *Slit1, 2*, and *3* mRNA. Slit 1, 2 and 3 expression was localized to the floor plate. Expression of *Slit2* and *3* co-localized to Islet 1/2+ neurons (D’, E’). Scale bars: B”, 50 μm, applies to Robo antibody labels; C’, 100 μm, applies to Slit in situs

During axon guidance, Slits derived from the floor plate act as repulsive signals via the Robo1 and Robo2 receptors. In this system, Robo receptors expressed on migrating cell bodies or neurites bind Slit ligands to signal repulsion away from the ventral midline see review on neural guidance in [34]. The Slit/Robo system was shown in the hindbrain to keep dorsal-projecting motor neuron axons out of the midline [23]. We considered whether floor plate-derived Slit may be in position to guide midline crossing of oculomotor cell bodies. Previous research in chick shows expression of *Robo*2 mRNA co-localized with the oculomotor nucleus early in development [35]. To determine the expression of Robo1 and 2 in mouse, we used immunofluorescence labeling on E10.5 for Robo1 and 2. Using primary antibodies against Robo1 or 2, we detected Robo1 and Robo2 antibody labeling co-localized to Islet1/2 positive cell bodies in the ventral midbrain, with varying levels of labeling throughout the nucleus (Fig. 3A, B). This indicates that both Robo1 and Robo2 are expressed by cells in the oculomotor nucleus. We also noted Robo1 and 2 antibody labeling of the exiting nIII axons (not shown), consistent with the cell body labeling.

Slit1 and 2 expression was previously shown to be prominent in ventral midbrain in E12 rat embryos [23], and in E10.5 mouse embryos [13]. To more specifically determine whether Slits are in a position to act as midline repellents prior to E13.5 in mouse, we examined the three Slits in the ventral midbrain on E10.5 by *in situ* hybridization of mRNA. We found *Slit 1, 2* and *3* mRNA was expressed in the ventral floor plate medial to the oculomotor nuclei, as well as a strip of cells in the underlying ventricular zone (Fig. 3C-E) on E10.5. Complementary expression of Robo receptors by oculomotor neurons and Slit expression in the floor plate supports a role for Slit repulsion from the floor plate, and may act to inhibit oculomotor migration prior to E13.5. The ventricular zone expression of Slits suggests a potential role in hemming the neurons into the marginal zone. We also found *Slit2* and *3* expression overlaps with the region of the Islet1/2-positive oculomotor neurons (Fig. 3D, E). This is consistent with Slit2 and 3 expression found in spinal motor neurons [36].

**Fig. 4:**
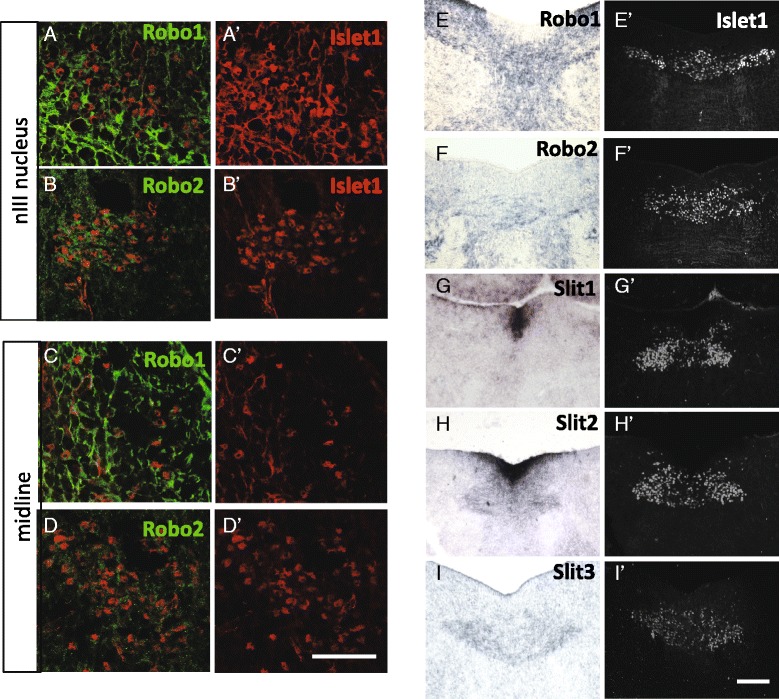
Slit and Robo remain in position to regulate floor plate crossing on E14.5. Coronal sections through the caudal oculomotor nucleus on E14.5 were labeled with Robo1 or 2 antibodies, or hybridized to Robo or Slit mRNA probes, followed by antibody labeling for Islet1/2 of the same section (for Robo antibodies), or adjacent sections (for in situ hybridization). **A**, **B** Within the nucleus, Robo1 and Robo2 was localized to Islet1/2+ cells indicating Robo expression by oculomotor neurons. **C**-**D**. In the midline of the caudal midbrain, Robo1 antibody labeling could be seen on some migrating neurons, while Robo2 labeling was less intense and variable. **E**, **F** In situ hybridization for Robo1 and 2 mRNAs showed labeling that overlapped with the nuclei, and also bridged across the midline in the area of migrating neurons. **G**-**I**. Slit mRNA expression by in situ hybridization, compared to Islet antibody labeling in adjacent sections. Slit1 and 2 continued to be expressed by floor plate cells on E14.5, while there was very little Slit3. *Slit2* and *Slit3* transcript was localized to the oculomotor nuclei as well as overlapping with the motor neurons migrating across the midline (**H**, **I**). Scale bars: D’, 50 μm, applies to Robo antibody labels; I’, 100 μm, applies to in situs

During oculomotor migration on E14.5, Robo1 and 2 protein remained expressed in the oculomotor nucleus (Fig. 4A, B). Although the expression levels appeared to vary across the nucleus, there was no clear pattern of different levels in lateral vs. medial/ventral areas of the nucleus (not shown). Robo1 and 2 labeling also appeared on oculomotor neurons migrating in the midline, although Robo2 levels were low and variable (Fig. 4C, D). To further confirm Robo expression during the migration phase, Robo1 and 2 mRNA domains also overlapped with the oculomotor nucleus in the ventral-caudal midbrain (Fig. 4E, F). Similarly, *Slit1* and *2* mRNA continued to be expressed in the ventral floor plate and ventricular zone tissue on E14.5 (Fig. 4G, H). In addition, Slit2 and 3 was expressed in a region that overlapped with the Islet1/2-positive oculomotor nucleus, including overlapping with the migrating population positioned in the midline (Fig. 4H, I). Thus, Slit1 and Slit2 are in position to act as midline repellents at the early stages of stationary motor neurons on E10.5, and are also maintained during migration on E14.5.

### Oculomotor cell bodies migrate prematurely in *Slit* and *Robo* mutant mice

**Fig. 5:**
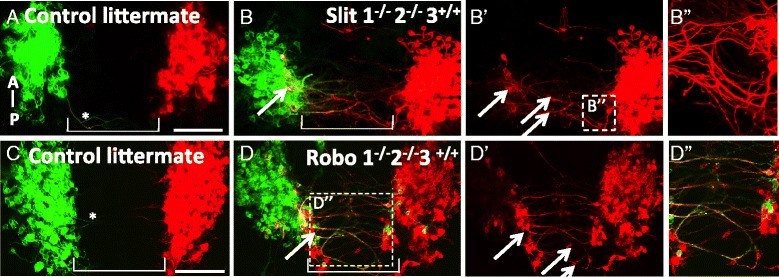
Motor neuron cell bodies migrate prematurely into and across the floor plate in Slit and Robo mutants. **A**-**D**. On E10.5, DiI and DiO were applied to the left and right (respectively) peripheral oculomotor nerves to back-label the oculomotor nucleus, as well as the leading process and somata directly connected to the peripheral nerve. Open book preparation of the mouse midbrain, with anterior up. **A**, **C** On E10.5 in wild type littermate controls, oculomotor somata and axons remained ipsilateral to the nucleus. Leading projections are rarely seen projecting from the oculomotor nuclei (**asterisk** in **A**, **C**). **B**, **D** *Slit1* ^-/-^,-2^-/-^ mutants or *Robo1* ^-/-^,2^-/-^ mutants had numerous leading processes projecting into and across the floor plate. Cell bodies were found in the ventral region of the contralateral nucleus (*yellow*, *arrows* in **B**, **D**). Bulges in leading processes appeared to be cell bodies migrating across the floor plate (arrows in B’,D’). Leading processes looped into and across the floor plate in Slit mutants (B”). Robo mutants displayed more fasciculation by leading processes traversing through the floor plate (*yellow color* in box (D”). (wild type, *n* = 6; *Slit1* ^-/-^, 2^-/-^, *n* = 8; *Robo1* ^-/-^,2^-/-^, *n* = 9) Scale bars, 100 μm

Slit expression in the ventral floor plate and Robo expression in oculomotor cell bodies suggests a function for floor plate-derived Slit in oculomotor migration. To determine whether Slit signaling mediates contralateral migration, oculomotor cell bodies were back-labeled from the oculomotor nerve(s) in Slit 1/2 or Robo1/2 double mutants and compared with wild type controls. In wild type controls on E10.5, three days prior to normal migration, the oculomotor nuclei were constrained to their location adjacent to the floor plate and ipsilateral to their corresponding nerve (Fig. 5A, C). Interestingly, in some control embryos on E10.5, rare cellular processes projected from the oculomotor nucleus, into the midline but did not appear to reach the contralateral nucleus (asterisk in Fig. 5A, C). These observations suggest a phase in normal development in which transient secondary processes are produced by early oculomotor neurons but are retracted and do not support the migration of cell bodies. However, in Slit1/2 or Robo1/2 double mutant embryos, numerous leading processes projected out from the nucleus reaching into the floor plate, with some reaching the contralateral nucleus (Fig. 5B, D). The path traveled by these processes was not linear, and frequently the processes curved back toward the nucleus of origin (Fig. 5B”). In Slit1/2 mutant embryos, we noted imprecise navigation by the leading processes, including looping and zig-zag patterns. Similar, but less numerous, loops were observed in Robo1/2 mutant embryos (Fig. 5D”). Therefore, the disruption of Slit/Robo signaling caused leading processes to project into and across the floor plate three days prior to normal migration.

**Fig. 6:**
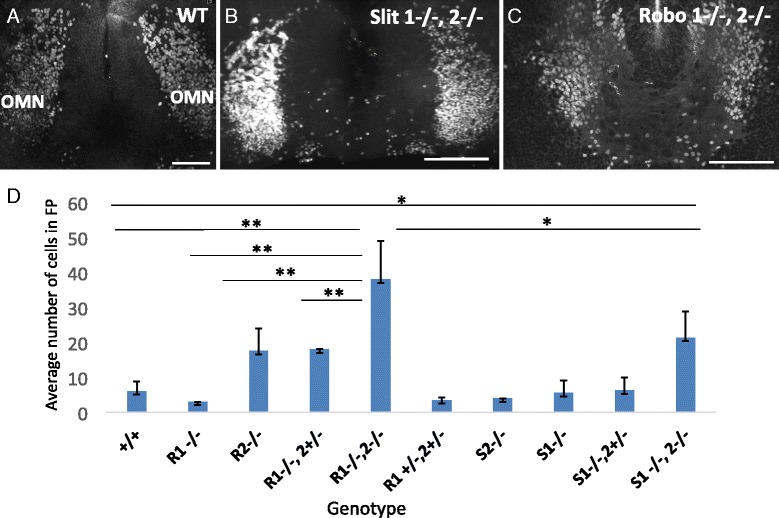
Cells migrating through the posterior midbrain in Slit and Robo mutants are motor neurons. **A**-**C** To identify and quantify migrating neurons that have separated from the oculomotor nucleus in Slit and Robo mutant mice on E10.5, open book preparations were antibody labeled with Islet1/2. In wildtype controls, few Islet1/2+ cells were found within the floor plate (**A**), while numerous Islet1/2+ cells were seen in the floor plate between the left and right oculomotor nuclei in both Slit and Robo mutants (**B**, **C**). **D** The average number of Islet 1/2+ cells located medial to the oculomotor nuclei were counted for each genotype (2 or more litters per genotype). The number of cells medial to the oculomotor nucleus in *Robo*1^-/-^,2^-/-^ and *Slit*1^-/-^, 2^-/-^ mutants is significantly more than wildtype controls. There are more Islet 1/2+ cells in the floor plate in *Robo* mutants compared to *Slit* mutants (**D**). Abbrev. Anterior (A), posterior (P). Scale bars, 100 μm, Error bars indicate standard deviation, **P < 0.01, *P < 0.05. (control, *n* = 5; *Robo1* ^-/-^, *n* = 2; *Robo2* ^-/-^, *n* = 4; *Robo1* ^-/-^,2^+/-^, *n* = 2; *Robo*1^-/-^,2^-/-^, *n* = 6; *Robo*1^+/-^, 2^+/-^, *n* = 5; *Slit*1^-/^, *n* = 2; *Slit*2^-/-^, *n* = 3; *Slit*1^-/-^,2^+/-^, *n* = 4; *Slit*1^-/-^,2^-/-^, *n* = 9)

Labeled neuron cell bodies in the ventromedial aspect of the contralateral nucleus as well as within several leading processes in Slit and Robo mutants on E10.5 suggest that cell bodies have crossed over from the opposing nucleus (arrow pointing to yellow color in Fig. 5B, D, arrows in Fig. 5B’, D’). To identify and quantify migrating cells, we labeled midbrain tissue for Islet1/2. In wild type controls, Islet1/2-positive cell bodies were rarely seen between the bilateral oculomotor nuclei (Fig. 6A). Conversely, in Slit and Robo mutant embryos, several Islet1/2-positive cells separated from the compact oculomotor nuclei, with several located within the floor plate (Fig. 6B, C). To determine the contribution of single or double Slit or Robo genes in midline crossing, we examined embryos mutant for single Slit or Robo genes (Fig. 6D). There was very little premature crossing observed in embryos homozygous mutant for Slit1, or embryos homozygous mutant for Slit2, in contrast to the strong crossing in Slit1/2 double homozygous mutants. Similarly, we found that a loss of one copy of *Robo1* did not increase Islet1/2 -positive cells in the floor plate. However, loss of Robo2 was sufficient to allow migration into the midline (Fig. 6D). Thus, early midline migration can be prevented by single functional Slit genes or a single functional Robo2 gene, suggesting redundant functions in midline crossing. The strongest effect was found in Robo 1/2 mutants, followed by Slit1/2 mutants. Interestingly, Robo1/2 mutant mice have twice as many Islet1/2 positive cells in the floor plate than Slit1/2 mutants. A more severe phenotype in Robo1/2 mutants compared to Slit1/2 mutant mice is consistent with previous suggestions of Slit-independent repulsive functions for Robo receptors [37].

### Loss of Slit 2 in motor neurons does not cause premature migration

**Fig. 7:**
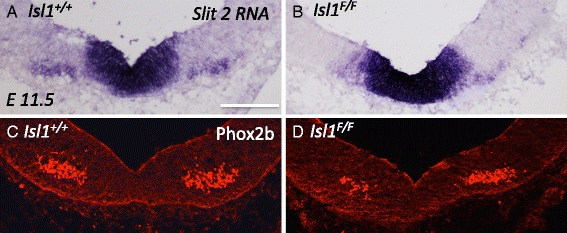
Loss of slit 2 in motor neurons is not sufficient to cause premature migration. **A**, **B** To confirm a loss of *Slit2* expression in the oculomotor nucleus in Islet1 mutant embryos, in situ hybridization was performed in the ventral midbrain on E11.5. **C**, **D**. Location of the oculomotor nucleus was determined by Phox2b expression (an Islet-independent transcription factor expressed in motor neurons). **A** In control embryos, *Slit2* RNA is found in the floor plate and co-localized to Phox2b positive cells (**C**). **B** In the Islet1 mutant midbrain, *Slit2* expression is retained in the floor plate, but is lost from the oculomotor nucleus (**D**). Phox2b positive neurons were clustered on either side of the floor plate, but not within the floor plate in both control and Islet1F/F mutants (**B**) indicating oculomotor cell bodies have not migrated into the floor plate (*n* = 4). Scale bar, 100 μm

From the initial discovery of mammalian Slit2 expression in the spinal cord floor plate, it was noted that spinal motor neurons also have cell-autonomous expression of Slit2 [36]. However, a cell-autonomous role for Slit2 in cell migration has not been examined. We also found Slit2 expressed by the oculomotor nucleus and midbrain floor plate cells on E10.5-E14.5 (Figs. 3 and 4). Because a global loss of *Slit* allows oculomotor neurons to migrate across the floor plate on E10.5 (Fig. 5), we wanted to separate the function of Slit2 derived from floor plate from Slit2 derived from the motor neurons. To determine whether Slit2 derived from oculomotor neurons plays a role in preventing premature migration on E11.5, we examined mice mutant for the gene Islet 1 (Isl1) that display a significant loss of *Slit2* in motor neurons [38]. We first confirmed a loss of *Slit2* in the oculomotor nucleus. In mice mutant for Isl1, there was little to no Slit2 transcript detected co-localized to the oculomotor nucleus, as counter-labeled with the alternative motor neuron marker, Phox2b (Fig. 7B). However, the strong midline Slit2 expression was retained. In mice mutant for Isl1, oculomotor neurons do not migrate across the midline prematurely (Fig. 7D). In addition, we confirmed in whole mount embryos that the oculomotor nerve forms its initial projections to the eye (data not shown). Thus, a loss of Isl1 and subsequently a loss of Slit2 (and potentially perturbed expression of other Islet-regulated genes) in oculomotor neurons does not result in premature migration. This is indirect evidence that suggests that premature migration in Slit or Robo mutant mice on E11.5 is due to a loss of Slit signals derived from the floor plate.

### The oculomotor commissure forms properly in Slit and Robo mutant mice

Oculomotor neuron migration across the midline on E10.5 in Slit and Robo mutant mice could be non-specifically affecting the entire nucleus, or could represent a premature but specific migration of those that normally migrate, that is, the superior rectus subset. Unfortunately, we were unable to identify a molecular marker for the superior rectus neurons in mouse embryos. However, we predicted that the oculomotor commissure would be larger if another population of cells migrated across the floor plate and joined the contralateral nucleus to augment the usual superior rectus commissural axon projection.

**Fig. 8:**
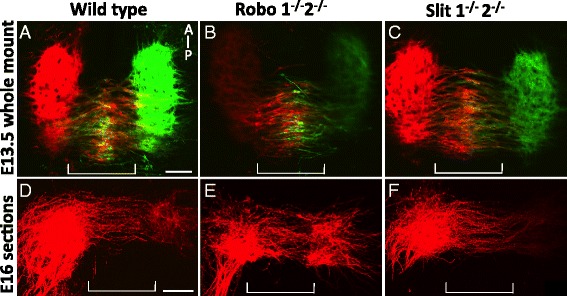
Slit and Robo mutants generate a normal oculomotor commissure. **A**-**C** Open book preparation of DiI and DiO back-labeled oculomotor nuclei. On E13.5, leading processes reached the contralateral nucleus. Neuron cell bodies, seen as bulges in the leading process, were in the midline (**A**, wild type control, Slit littermate). Loss of Slits or Robos (**B**, **C**) did not appear to reduce the number of leading processes or cells migrating through the floor plate. **D**-**F**. DiI back-label of the oculomotor nucleus on E16. 200 um coronal sections along the plane of the oculomotor nerve were compared by z-stacked confocal images. The oculomotor commissure was similar in thickness to wild type (**D**) in Slit and Robo mutants (**E**, **F**). (*n* = 3; control, Robo and Slit mutants) Scale bars, 100 μm

We examined Slit and Robo null mutant embryos during leading process extension and cell migration on E13.5, and on E16, after migration ceased. In wild type embryos on E13.5, leading processes projected into and across the floor plate. Midline crossing was restricted to the caudal half of the nucleus. Migrating oculomotor cells could be seen in the midline, and approaching the ventral region of the contralateral oculomotor nucleus (Fig. 8A). Both Slit and Robo mutant mice had oculomotor neurons that appear similar to wild type, with leading processes and cell bodies located within the floor plate on E13.5 (Fig. 8B, C). However, we note that Robo1/2 mutant commissures had a more disordered appearance than Slit1/2 mutant commissures. It appeared in some cases that the Slit double mutant commissure may contain more axon fibers, and possibly extend further rostrally. However, because of the inability to quantify the inherently variable back-labeling tracing strategy, and the lack of a specific molecular marker for superior rectus neurons, we could not definitively distinguish whether ectopic oculomotor neurons were recruited to cross the midline.

To determine if Slit and Robo influence the final development of the commissure on E16.5, DiI-labeled oculomotor nuclei were sectioned across a coronal plane through the commissure. In controls, the oculomotor commissure was fully developed with leading processes, spanning the floor plate (Fig. 8D). DiI-labeled processes intercalated throughout the contralateral nucleus. No cell bodies were seen within the floor plate, suggesting that the contralateral migration was complete by E16.5 (Fig. 8D). In E16.5 Slit1/2 and Robo1/2 mutants, the oculomotor commissure was positioned caudally, as in controls, with a similar size of the commissure (Fig. 8E, F). Together, this data suggests that premature migration on E10.5 does not appear to influence normal migration at later stages.

### Regulators of Slit signaling are not required for contralateral migration

Migration of oculomotor cells on E10.5 in Slit and Robo mutant embryos suggests a mechanism in which wild type oculomotor neurons are initially trapped in position by Slit/Robo repulsion, but later, on E13.5, suppression of Slit/Robo signals allow for migration into and across the midline. We considered and tested three mechanisms that suppress Robo repulsive signaling in other systems.

**Fig. 9:**
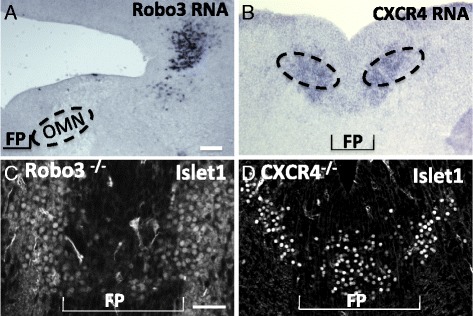
Robo3 or CXCR4 function is not required for oculomotor migration into the floor plate. **A**, **B**. To determine if Robo3 or CXCR4 are in position to repress Slit/Robo signaling in situ hybridization of RNA was performed on E14.5 midbrain sections. Robo3 was not expressed in the ventral midbrain on E14.5 (**A**). However, CXCR4 was expressed in the ventral midbrain in both the floor plate and regions lateral to the floor plate (**B**). **C**, **D** To identify migration patterns of oculomotor neurons on E13.5 in Robo3 and CXCR4 mutants, sections of the posterior midbrain were antibody labeled with Islet1/2. Islet1/2+ cells migrated into the floor plate in both Robo3 (**C**) and CXCR4 mutants (**D**) indicating neither Robo3 nor CXCR4 are required for migration oculomotor migration across the floor plate. (Robo3, *n* = 2; CXCR4, *n* = 4) Scale bar, 100 μm

In the first Slit/Robo suppression mechanism, we examined Robo3. Robo3 has been proposed to act as a negative regulator of Slit repulsion via dominant negative action on Robo1 and Robo2. In the spinal cord, Robo1 and 2-expressing pre-crossing spinal cord commissural axons are allowed to approach the floor plate when Robo3 is co-expressed allowing these axons to enter an area of high Slit expression [15, 39]. Alternatively, Robo3 may potentiate Netrin attraction to counteract Slit repulsion [16]. We therefore hypothesized that Robo3 might be expressed on oculomotor neurons on E13.5 to negate Slit repulsion from the floor plate. *In situ* hybridization against *Robo3* mRNA on E13.5 showed that *Robo3* expression was restricted to a subset of neurons found just dorsal to the OM nucleus (Fig. 9A). To formally rule out a requirement for Robo3, we examined whether oculomotor neurons migrated across the floor plate in mouse embryos lacking Robo3, and found Islet-positive cells in the midline on E13.5 (Fig. 9C). This suggests that Robo3 activity is not required for oculomotor migration into or across the floor plate.

A second potential antagonist of Slit signaling is the receptor CXCR4. In chick, the binding of SDF-1 to CXCR4 suppresses the repellent activity of Slit2, Semaphorin3A, and 3C on axons cultured in vitro [40]. In zebrafish retinal ganglion cells, SDF-1 antagonizes Slit/ Robo2 signaling in vivo [41]*.* CXCR4 is expressed in the mouse ventral midbrain where it is required for the exit of oculomotor and other cranial nerves [42]. We confirmed CXCR4 expression in the oculomotor nucleus by *in situ* hybridization (Fig. 9B). Therefore, we were interested in whether floor plate CXCR4 may antagonize Slit signaling in migrating oculomotor neurons to allow for midline crossing. However, in E13.5 CXCR4 mutant embryos, Islet1/2-positive cells were found in the midline (Fig. 9D). This suggests that CXCR4 signaling is not required to antagonize the repellent activity of the floor plate.

### Netrin1 attraction is not required for oculomotor migration into the floor plate

**Fig. 10:**
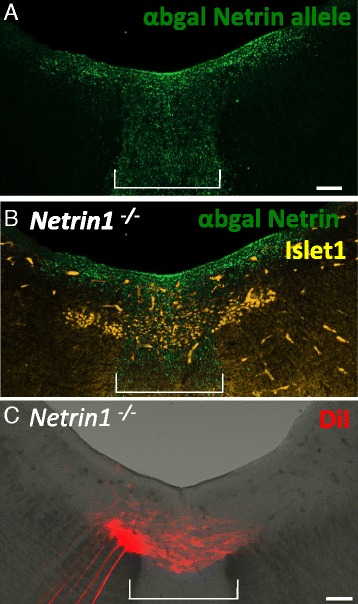
Netrin1 is not required for oculomotor migration into the floor plate. **A**, **B** To locate Netrin expression during oculomotor migration, the mutant allele Netrin1^lacZ^ was labeled with anti beta-gal in Netrin1-/- (i.e. lacZ/lacZ) mutant mice. On E13.5, Netrin1 was expressed by the floor plate and ventricular layer of cells in ventral midbrain (**A**), adjacent to Islet1/2+ motor neurons located in the nucleus and in the floor plate (**B**). **C**. DiI back-label from nIII in a Netrin1^-/-^ embryo on E13.5. A 200 um coronal section along the plane of the oculomotor nerve was imaged by z-stacked confocal images. Leading processes projected from the oculomotor nucleus toward the contralateral nucleus (*n* = 3). Scale bar, 100 μm

Rather than blocking a repulsive signal, the prematurely migrating cell bodies could be activating a response to a floor plate-derived attractant. In chick, overexpression of the N-terminal domain of the actin-binding protein Drebrin caused leading processes to orient toward the trochlear nucleus instead of the contralateral oculomotor nucleus [6]. This study suggests that leading processes are initially attracted toward the floor plate. The classical attractant found in the floor plate is Netrin1. In the hindbrain, Netrin1 is required to attract precerebellar neurons toward the floor plate [17]. Netrin/DCC signaling also attracts basal pontine neurons toward the ventral midline [43]. Interestingly, a previous study showed that rat ventral midbrain explants produced neurites that were repelled by floor plate tissue, but were unresponsive to Netrin1-secreting cell aggregates, although the experiment did not distinguish whether the responding neurites were primary motor axons or midline crossing leading processes [22]. We therefore examined *Netrin1* expression on E13.5. We find *Netrin1* expression in the ventricular surface and floor plate in the midbrain (Fig. 10A). However, in Netrin1 mutant mice, we find Islet1/2-positive cells in the floor plate on E13.5 (Fig. 10B), as well as normal projections toward the contralateral nucleus (Fig. 10C). Therefore, Netrin1 signaling is not required to attract oculomotor neurons or their leading processes toward the floor plate.

### Contralateral OM migration is regulated by signals intrinsic to the ventral midbrain

The initiation of contralateral migration coincides with the time when the oculomotor axons reach the superior rectus extraocular muscle primordia in chick [5, 44, 45]. It has therefore been proposed that a signal from a peripheral target may initiate migration [44]. To determine whether a signal is transported from the developing extraocular muscles back to the cell body to initiate migration, we utilized three techniques to eliminate interaction between the oculomotor nerve and the peripheral tissue, and examined oculomotor neuron migration.

**Fig. 11:**
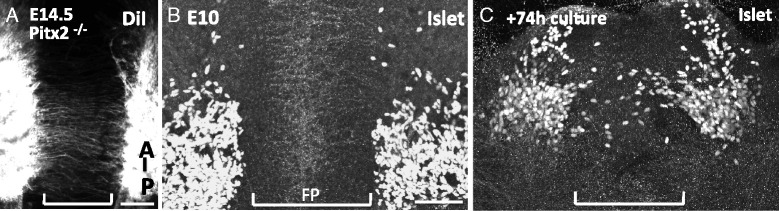
Oculomotor midline migration is independent of peripheral signals. **A**. Open book preparation of the midbrain in a mouse lacking extraocular muscles in Pitx2 mutant mice, with the oculomotor nucleus back labeled with DiI. On E14.5, a commissure originating from the posterior half of the oculomotor nucleus crossed the floor plate (*n* = 3). **B**, **C**. Explant culture of isolated midbrain tissue. E10.5 midbrains were dissected to remove all peripheral tissue, including nIII, then cultured in an open book preparation in collagen gel for 72 h. Cultured tissue was then labeled with Islet1/2 antibody. Anterior is up; floor plate indicated by bracket. **B**. Dissected tissue at the onset of culture period showed Islet1/2+ cell bodies on either side of the floor plate. **C**. Following incubation for 72 h, a posterior subset of oculomotor neurons migrated into the midline (*n* = 4). *Brackets* indicate floor plate region. Scale bars, 100 μm

In the first approach, we examined mice mutant for the transcription factor *Pitx2,* where extraocular muscle precursors undergo apoptosis prior to the nerve reaching the precursor cells [46]. We first confirmed that Pitx2 mutant mice lacked muscles on E14 in both sagittal and coronal sections (data not shown). To determine whether an oculomotor commissure develops in mice lacking extraocular muscles, we back-labeled both left and right oculomotor nuclei with DiI from nIII. The oculomotor commissure is apparent in these mice and originates from the posterior half of the nucleus (Fig. 11A). Therefore, signals derived from the extraocular muscles are not required to initiate migration.

To eliminate the possibility that peripheral signals derived from tissue other than the extraocular muscles initiate midline migration, we utilized a second in vitro technique where the midbrain was dissected away from the oculomotor nerve and peripheral tissues on E11.5. The midbrain, devoid of peripheral tissue, was cultured for 72 h in vitro, then oculomotor neurons were labeled*.* At the starting point on E11.5 (0 h), all oculomotor neurons reside on the edge of the floor plate with no Islet1/2-positive neurons in the floor plate (Fig. 11B). Following 72 h in vitro, Islet1/2-positive neurons were found throughout the floor plate, particularly in the posterior half of the nucleus where migration normally occurs in vivo (Fig. 11C). Therefore, isolated midbrain cultures provided sufficient cues to initiate and guide migration, and demonstrate that removal of the peripheral nerve does not appear to restrict oculomotor migration.

A caveat to the explant culture strategy was that the initial outgrowth of the nerve toward the eye occurs on E9.5. Therefore, a signal to activate migration could be transported back to the oculomotor nucleus prior to E11.5, although it must remain latent until overt migration begins on E13.5. To test the possibility that a signal is transported during the initial outgrowth of nIII, we examined a mutant mouse where the oculomotor nerve outgrowth into the periphery is absent. In CXCR4 mutant mice, axons projecting from the oculomotor nucleus wander dorsally within the neuroepithelium instead of toward the peripheral mesenchyme where the attractive CXCR4 ligand, SDF-1, is expressed [42]. These mutants have an oculomotor nerve that aberrantly projects within the neuroepithelium, or is much smaller in size [42]; therefore, nIII would be unable to obtain or transport peripheral signals back to the nucleus to initiate migration. We first verified that the oculomotor nerve in CXCR4 mutant mice was missing, both by visual inspection during dissection and axon antibody labeling in whole mount embryos. We were unable to identify a peripheral oculomotor nerve emerging from the midbrain (data not shown), confirming the previous report, and suggesting that a signal from a peripheral intermediate target was unlikely to be transported back to the nucleus in CXCR4 mutant mice. As previously stated, we found Islet1/2-positive cell bodies in the floor plate of the midbrain in CXCR4 mutant mice (Fig. 9D), indicating that contralateral oculomotor migration can occur independent of nerve-derived signals obtained from outside of the neural tube. This suggests that signals to initiate migration are intrinsic to the oculomotor nucleus and nearby tissues within the ventral midbrain.

## Discussion

Biondi first observed a group of neurons that separate from the developing oculomotor column and migrate toward the opposing oculomotor nucleus [47]. Later, through a series of Golgi labels, Puelles proposed that “diffusible factors” are likely responsible for guiding migration [3]. Until our study, the identity of such factors remained unknown.

To initiate and guide the several steps involved in migration across the midline, we speculate that a combination of diffusible extrinsic signals and intrinsic factors are required. The migration of a subset of oculomotor neurons appears to involve multiple steps: 1) the superior rectus motor neurons receive extracellular or intracellular signals to initiate and extend a secondary leading process; 2) leading processes receive guidance signals to attract them toward the midline and eventually toward the contralateral nucleus; 3) the neuron cell bodies lose cell adhesion to the ipsilateral oculomotor nucleus and follow their secondary leading processes across the floor plate; 4) neurons must move to and integrate into the contralateral nucleus.

Here we show that Slit signaling is necessary to inhibit the initiation of oculomotor migration. Slits may function to gate migration, such that the suppression of Slit/Robo repulsion on E13.5 would allow a subset of oculomotor neurons to turn on migratory processes. Our data suggest a range of potential roles for Slit signaling in migratory OM neurons including; blocking extension of leading processes, promoting cell adhesion to ipsilateral nucleus, and blocking migration of the neuronal cell bodies across the floor plate.

### Migrating oculomotor neurons pioneer an independent path across the midline via leading axon-like processes

Oculomotor neurons initially make a conventional axon projection that exits the CNS out toward their peripheral targets. However, then a subset undergo a remarkable transition to produce second axon-like fibers (leading processes) oriented toward and across the ventral midline, followed soon after by translocation of the neuronal cell bodies [5]. We confirmed this time course in the mouse: all migrating oculomotor neurons possess a leading process that projects toward the midline, with a trajectory that is perpendicular to the radially-oriented ventricular cells in the floor plate on E13.5. The leading processes originate only from neurons in the caudal half of the nucleus, and can extend to reach the opposing oculomotor nucleus. These processes appear similar to leading processes found in migrating pontine neurons [43]: leading processes are long, branching, and fasciculate with other processes.

Similar to pontine neurons, the guidance of oculomotor leading processes does not depend upon pre-existing glial structures [2] or the existing tecto- tegmental commissure located in the floor plate. A distinct gap between the existing tecto-tegmental commissure and migrating motor neurons can be found in every section through the oculomotor nucleus. Therefore, oculomotor leading projections must navigate using other environmental cues to reach their target, and suggests that they pioneer a path through a permissive corridor across the midline, independent of the tegmental commissure. The potential molecular and cellular substrates for the oculomotor commissure remain undefined.

The observation in the midline of both cell bodies and secondary leading processes suggested that contralateral cell bodies were not the result of their primary axons making midline crossing errors. The number of migrating motor neurons is surprisingly large, as in posterior sections they outnumber the remaining non-migratory neurons. Interestingly, a large degree of cell death in the ventromedial aspect of the chick oculomotor nucleus was previously noted [48]. This suggests a large proportion of migratory neurons initiate migration but later succumb to cell death.

### Slit signals prevent premature migration of oculomotor neurons

Guidance cues of the Slit family have emerged as repulsive regulators of midline crossing of axons and, more recently, in neuron cell bodies in both vertebrates and invertebrates reviewed by [49]. In the ventral midbrain on E10.5, both Slit1 and 2 are expressed in the floor plate, and the Slit receptors, Robo1 and 2, expressed by the oculomotor neurons. Slit1 and 2 signals from the floor plate may actively repel the leading process, and hence oculomotor neurons, away from the floor plate via the Robo receptors. Consistent with this idea, we show significant premature outgrowth of leading processes in Slit1/2 or Robo1/2 mutants on E10.5. This is coupled with oculomotor cell migration across the floor plate in a subset of cells in the posterior region of the oculomotor nucleus. Previous research describes a similar role for Slit midline repulsion in the hindbrain where Slit1 and 2 are required to keep dorsally projecting motor neurons, but not ventrally projecting nerves, out of the floor plate [23]. We also find that Slits are not required to guide the ventrally projecting nerve fibers, but are required to inhibit the growth of leading processes and migration of cell bodies across the floor plate. In chick, outgrowth of leading processes depends upon the actin-binding protein Drebrin [6]. Therefore, Slit signaling may inhibit Drebrin activity to repress leading process outgrowth prior to E11.5. However, an interaction between Slit/Robo and Drebrin has yet to be described.

We found Slit2 and Slit3 transcripts in the oculomotor nucleus on E10.4 and E14.5. Slit2 and Slit3 also appear to be expressed by other motor neurons [36]. In motor neurons, it is suggested that Slit2 and Slit3 act cell autonomously to modulate their own Robo receptor responsiveness [36], or are transported to the axon to promote nerve fasciculation [50]. Our data from Isl1 mutant mice suggests that motor neuron-derived Slit2 does not participate in repelling oculomotor neurons from the floor plate on E11.5. However, Slit2 cell autonomous functions in the oculomotor nucleus remain to be clarified by future experiments with motor neuron-specific knockouts.

We find an increased number of cell bodies migrating across the midline in Robo mutants compared to Slit mutants on E10.5. This suggests that Robo may have additional ligands that mediate cell adhesion or inhibit cell migration. Signaling from the Robo receptor modulates cell adhesion by N-cadherin [51], and acts to either increase or decrease adhesion [52]. However, cell adhesion in these systems is dependent upon the Slit ligand. Future research may give insight into how the Robo receptor differentiates between mediating cell adhesion and signaling repulsion.

Taken together, we propose that Slit/Robo signals keep leading processes away from the floor plate, and also possibly inhibit neural translocation within the processes. In a similar case, precerebellar neurons of the inferior olive (IO) respond to repulsive Slit signals from the floor plate. IO neurons normally project a leading process across the midline, but their cell body stops just prior to entering the floor plate in the hindbrain. In Robo1/2 mutant mice, IO cell bodies migrate across the floor plate, indicating that Slits are acting as midline repellents via the Robo1/2 receptors [53, 54]. The leading processes in the IO are not normally repelled from the midline, indicating that these two actions—leading process guidance and neural translocation— are independent of each other, with neural translocation being susceptible to repellent Slit signals in IO neurons. However, in the oculomotor system, both leading process outgrowth and neural translocation appear equally responsive to repellent Slit signals.

Migration into the floor plate on E10.5 in Slit and Robo mutants is seen in only a subset of oculomotor neurons. These neurons emanate from the caudal half of the ipsilateral nucleus and are competent to cross the floor plate to reside in the contralateral nucleus. Wild type migration of superior rectus motor neurons occurs similarly, starting with a leading process projecting from the caudal half of the ipsilateral nucleus toward the contralateral nucleus, neuronal migration, and finally integration into the contralateral nucleus. The caudal location and size of the commissure suggests that oculomotor neurons that migrate on E10.5 in Slit and Robo mutants are superior rectus motor neurons that migrate prematurely. Premature projection of retinal ganglion cell axons also occurs in Slit1 and 2 mutant mice [12]. Premature migration suggests that superior rectus motor neurons are responsive to Slit repulsion at the floor plate prior to E13.5 and that Slit/Robo repulsion keeps the motor neurons in their initial ipsilateral positions until this later time point. However, a method to specifically label superior rectus motor neurons is not currently available, so we were not able to distinguish which sub-populations of oculomotor neurons were recruited to cross the midline in Slit or Robo mutants.

### Factors that regulate the timing of Slit repulsion of motor neurons

We suggest that Slits initially repel superior rectus motor neurons on E10.5. Later, on E13.5, this subset must become insensitive to Slit repulsion to enter the floor plate. This could occur by blocking the repulsive activity of floor plate-derived Slit, or increasing oculomotor attraction to the floor plate. Turning off the Slit signal could occur by a general decrease of Robo on the surface of migrating neurons. For example, in *Drosophila*, the cytoplasmic protein Commissureless acts to reduce Robo receptors at the cell surface thereby inhibiting repulsive Slit signaling [55, 56]. However, Commissureless homologues have not yet been identified in vertebrates, so it remains unclear whether an analogous mechanism might be involved with vertebrate Robos.

Activating receptors that compete with, or block Slit/ Robo signaling, may be another strategy to release neurons to migrate into Slit positive territory. We tested the possibilities that receptors Robo3 or CXCR4 may interfere with Slit signaling allow for Robo-expressing oculomotor neurons to migrate into zones of high Slit expression. Although some evidence suggested that oculomotor neurons express Robo3 and CXCR4, we found that these receptors are not required for midline crossing. We also found that Netrin1 expressed by the ventral floor plate is not required for migrating oculomotor neurons. This may indicate that other attractive proteins function to attract oculomotor neurons either in concert with Netrin1 or independently. Other signals that block Slit/Robo signaling include NPN1. NPN1 interacts with Robo1 to reduce the repulsive effects of Semaphorin3A in cortical interneurons [57]. In chick, class 3 Semaphorins are expressed in the oculomotor nucleus as well as in the ventral floor plate, while NPN1 is found in migrating oculomotor neurons [44]. Therefore, NPN1 receptors may interact with Robo receptors on superior rectus motor neurons to allow for migration. However, we were unable to detect NPN1 antibody labeling in the oculomotor nucleus (data not shown). Future work may investigate the contribution of Semaphorin and Neuropilin signaling in mediating oculomotor migration.

### Oculomotor migration does not rely on signals from target tissues

The coincidental timing of superior rectus extraocular muscle innervation and motor neuron migration suggests that a signal obtained from the muscle or intermediate target may be transported from peripheral tissue to the cell body to initiate migration [44]. However, we found that oculomotor neurons migrate in the absence of extraocular muscles, peripheral tissue, and the peripheral nIII itself. These results agree with previous findings that superior rectus motor neurons migrate in Phox2b knock-in mice that lack the oculomotor nerve [58]. Signals from within the neural tube therefore provide the signal to initiate migration. For example, the idea that the tecto-tegmental commissural axons traveling from the dorsal midbrain toward and across the floor plate may initiate oculomotor migration [59] was tested by ablation of the commissural axons during chick development. However, oculomotor neuron migration was not altered [60]. Likewise, it was suggested that dopamine generated by midline cells that later generate the substantia nigra and ventral tegmentum may attract oculomotor neurons [3]. However, there is no evidence thus far that monoamines have chemoattractant properties.

Therefore, other signals from within the neural tube, likely in conjunction with intrinsic properties such as cell surface receptors, specific to superior rectus motor neurons, may initiate the outgrowth of the leading projection and subsequent neural migration.

## Conclusion

Migration of superior rectus motor neurons across the ventral midline to the contralateral oculomotor nucleus likely requires tight regulation by a number of extrinsic cues and intrinsic responses. Mice lacking *Slit1* and *2* or *Robo1* and *2* have motor neurons that migrate to the contralateral nucleus on E10.5, three days prior to the normal migration on E13.5. This suggests active Slit/Robo repulsion from the midline is required, at least at early stages, to maintain the ipsilateral position of oculomotor neurons. Neurons that migrate on E10.5 in Slit1/2 or Robo1/2 mutants are positioned in the caudal half of the nucleus, suggesting these neurons are superior rectus motor neurons migrating prematurely. Migration across the midline at E10.5 requires competency to interpret midline attraction, as well as attraction toward the contralateral nucleus. The classic midline attractant Netrin1, or its receptor DCC, does not appear to be required for successful superior rectus motor neuron migration. However, other attractive signals working independently of, or redundant to, Netrin1 are available as early as E10.5 to promote midline crossing in *Slit* and *Robo* mutant mice. Furthermore, the ability to respond to and initiate migration across the midline is intrinsic to the neural tube, and does not require signals obtained from the peripheral tissue.

## Abbreviations

DCC: Deleted in cortical cancer
DiI,DIO: Long Chain Dialkylcarbocyanine, Lipophilic Dyes
E: Embryonic day
IO: Inferior olive
Isl1: Islet 1
mlf: Medial longitudinal fasciculus
mRNA: Messenger ribonucleic acid
nIII: Cranial nerve three
NPN1: Neuropilin 1
OMN: Oculomotor nucleus
PBS: Phosphate buffered saline
PBST: Phosphate buffered saline with triton detergent
SHH: Sonic hedge hog
TC: Tegmental commissure
